# Comparison of plantar pressure distribution between three different shoes and three common movements in futsal

**DOI:** 10.1371/journal.pone.0187359

**Published:** 2017-10-31

**Authors:** Meghdad Teymouri, Farzin Halabchi, Maryam Mirshahi, Mohammad Ali Mansournia, Ali Mousavi Ahranjani, Amir Sadeghi

**Affiliations:** 1 Department of Physical Education, Payam Noor University (PNU), Shahrood, Iran; 2 Sports Medicine Research Center, Tehran University of Medical Sciences, Tehran, Iran; 3 Department of Sports and Exercise Medicine, Imam Khomeini Hospital Complex, Tehran University of Medical Sciences, Tehran, Iran; 4 Sina Hospital, Tehran University of Medical Sciences, Tehran, Iran; 5 Department of Epidemiology & Biostatistics, Tehran University of Medical Sciences, Tehran, Iran; 6 Faculty of Engineering, Islamic Azad University-South Tehran Branch, Tehran, Iran; 7 Faculty of Engineering, Rozbahan University, Sari, Iran; Universita degli Studi di Verona, ITALY

## Abstract

**Introduction:**

Analysis of in-shoe pressure distribution during sport-specific movements may provide a clue to improve shoe design and prevent injuries. This study compared the mean and the peak pressures over the whole foot and ten separate areas of the foot, wearing different shoes during specific movements.

**Methods:**

Nine male adult recreational futsal players performed three trials of three sport-specific movements (shuffle, sprint and penalty kick), while they were wearing three brands of futsal shoes (Adidas, Lotto and Tiger). Plantar pressures on dominant feet were collected using the F-SCAN system. Peak and mean pressures for whole foot and each separate area were extracted. For statistical analysis, the mean differences in outcome variables between different shoes and movements were estimated using random-effects regression model using STATA ver.10.

**Results:**

In the average calculation of the three movements, the peak pressure on the whole foot in Adidas shoe was less than Lotto [8.8% (CI95%: 4.1–13.6%)] and Tiger shoes [11.8% (CI95%:7–16.7%)], (P<0.001). Also, the recorded peak pressure on the whole foot in penalty kick was 61.1% (CI95%: 56.3–65.9%) and 57.6% (CI95%: 52.8–62.3%) less than Shuffle and Sprint tests, respectively (P<0.001).

**Conclusion:**

Areas with the highest peak pressure during all 3 movements were not different between all shoes. This area was medial forefoot in cases of shuffle and sprint movements and medial heel in case of penalty kick.

## Introduction

Futsal (indoor soccer) is an increasingly growing sport all over the world. According to FIFA Big Count, it is played by over one million registered players worldwide [1]. Futsal is characterized by frequent movements including accelerating and stopping, cutting, jumping, kicking, and tackling [2]. As a result, physical demands are very high [3–5].

Epidemiological studies report a high incidence of lower limb overuse injuries, owing to playing characteristics and hard field surface [2,6,7]. It seems that overuse injuries of foot and ankle are very common in futsal [8,9]. Diverse extrinsic and intrinsic risk factors may play a role in the etiology of these injuries [10–13]. Extrinsic risk factors are related to factors outside human body, such as exercise load, environmental conditions, as well as surface and equipment used. [14]. Shoe is an extrinsic factor and may influence the risk of overuse injuries [11–13]. Due to significant technical dissimilarities, soccer and futsal shoes have relatively different designs. Compared to soccer, futsal players use kicking techniques differently; for kicks on goal, they shoot much more often by the instep and toe poke, whereas in soccer the full instep is used most frequently. Furthermore, futsal players control the ball in most cases with the outsole on the bottom of the shoe. In soccer, however, the inside of the shoe is used most regularly [15]. Accordingly, futsal shoes are usually extremely lightweight. They have low profile rubber outsoles for traction on flat indoor courts and turf surfaces.

Despite the importance of shoe shock absorption and plantar pressure in overuse injuries, there is no quantitative data available regarding the foot-loading characteristics during futsal-specific movements. Knowledge about the amount and distribution of plantar pressure is valuable for the development of specific shoe/insole designs and also the prevention of overuse injuries [11,16]. However, a few scientific studies have been published on this issue. Plantar pressure measurement provides clinicians and researchers with practical information regarding the structure and function of the foot, general biomechanics of gait and is a valuable tool to assess patients with foot injuries [17]. There are two main systems to measure plantar loading pressure: platform devices placed between barefoot and floor and in-shoe sensor systems placed between the foot and the shoe. It seems that in-shoe techniques may be advantageous because they monitor the most important interface (between foot and shoe) and allow increased flexibility of measurement and more vigorous statistical analysis [16]. However, plantar pressure pattern data are not generally easy to analyze and therefore difficult to interpret. In most cases, mean and peak integrals of plantar pressure are used [18–19]. Thus far, foot has been divided into anatomical areas to calculate plantar pressure parameters. Generally, six to eleven of such areas are considered [19–20]. Information on the pressure distribution in different foot areas during weight bearing movements is considered to be important for the practice of sports medicine [21]. Various methods have been used for estimation of gait parameters; however, only few can provide reliable information on foot pressure distribution during gait and movement. Recently, pressure sensitive in-sole system has gained more popularity and is advocated by some researchers [22–23].

The aim of the present study was to investigate and compare the mean and peak pressures over whole foot and different areas of the foot analyzing three different futsal shoes during three sports specific movements. Our hypothesis was that the pattern of plantar pressure distribution may change according to the movement or shoe type.

## Material and methods

First, 12 right foot-dominant adult recreational futsal players with shoe size of 42 were assessed for common malalignments such as foot hyperpronation, Genu varum or valgum, hip anteversion and hallux valgus and recent injuries in lower extremity (during the last 6 months). Due to these disorders, three players were excluded from our study. The remaining nine players [Age (year): 22.6 (2.7)] then participated in an educational session before the testing procedures. Then, some anthropometric parameters such as weight, height, foot length (distance between two parallel) lines that are perpendicular to foot and in contact with the most prominent toe and the most prominent part of the heel) and foot width (widest part of foot) were measured [height (cm): 173 (7.1), weight (kg): 71.1 (6.6), body mass index (kg/m^2^): 23.8 (2.3), foot length (cm): 25.8 (0.7) and foot width (cm): 10.1 (0.7)] and participants performed three trials of three sport specific movements wearing three models of futsal shoes (Adidas Predator 2011, Lotto Puntoflex 2011, and Tiger Original 2012).

Specific futsal movements of this study include Shuffle/CUT45, Sprint, and Penalty kick test. Therefore, 243 foot pressure measurements were recorded (9 participants * 3 different movements * 3 trials * 3 different shoes).

For the shuffle/CUT 45 maneuver, a 10-meter wide and 20-meter long area was prepared. Five cones with a distance of 10 meters were used. The subjects were instructed to sidestep to the left side as fast as possible across a 10-meter path (from the first cone to the second cone), change direction to the right side at the 45-degree angle (CUT 45) and run to the third cone, change the direction again to the left side at the 45-degree angle (CUT 45) and run as fast as possible to the fourth cone, and alter the way and sidestep to the right side to the last cone. For the sprint maneuver, each player ran over a distance of 20 meters with his peak effort. In the penalty kick maneuver, subjects performed three individual steps in an approach before kicking the ball. Pressure distribution was collected under the dominant foot for shuffle and sprint maneuvers and supporting (stance) foot for penalty kick. To obtain enough measurements, each movement was performed at least three times. The first trial was only for players’ familiarity, the second and the third trial were for pressure measurement, and the fourth trial was performed in cases where there was more than 20% difference of recorded mean or peak pressures between the second and the third trials.

The Ethical Committee of Tehran University of Medical Sciences approved the study protocol according to recommendations of the declaration of Helsinki. All participants signed a written informed consent before entering the study.

Athletes’ plantar pressures were collected during the selected common maneuvers of futsal wearing the three different brands of futsal shoes (Adidas, Lotto, and Tiger) using the F-SCAN system (Tekscan, South Boston, MA, the USA) with methods previously described and validated. [21] Each pressure sensor insert was 0.38 mm thick, contained 884 sensors (in a 52 ˣ 17 matrix), and measured pressure over a range of 0–862 kPa. A pressure sensor fitted to subjects’ shoe size (European size 42 or American size 9 in all), was placed inside the shoes. The sensors were connected to a computer laptop via a 16-meter long cable. This cable was taped and secured to the waist of the participants so that no limitation or barrier would disturb their movement. The sensors were calibrated in the beginning and midway through the test or as needed, according to the manufacturer guidelines and standardized techniques.

**Fig 1:**
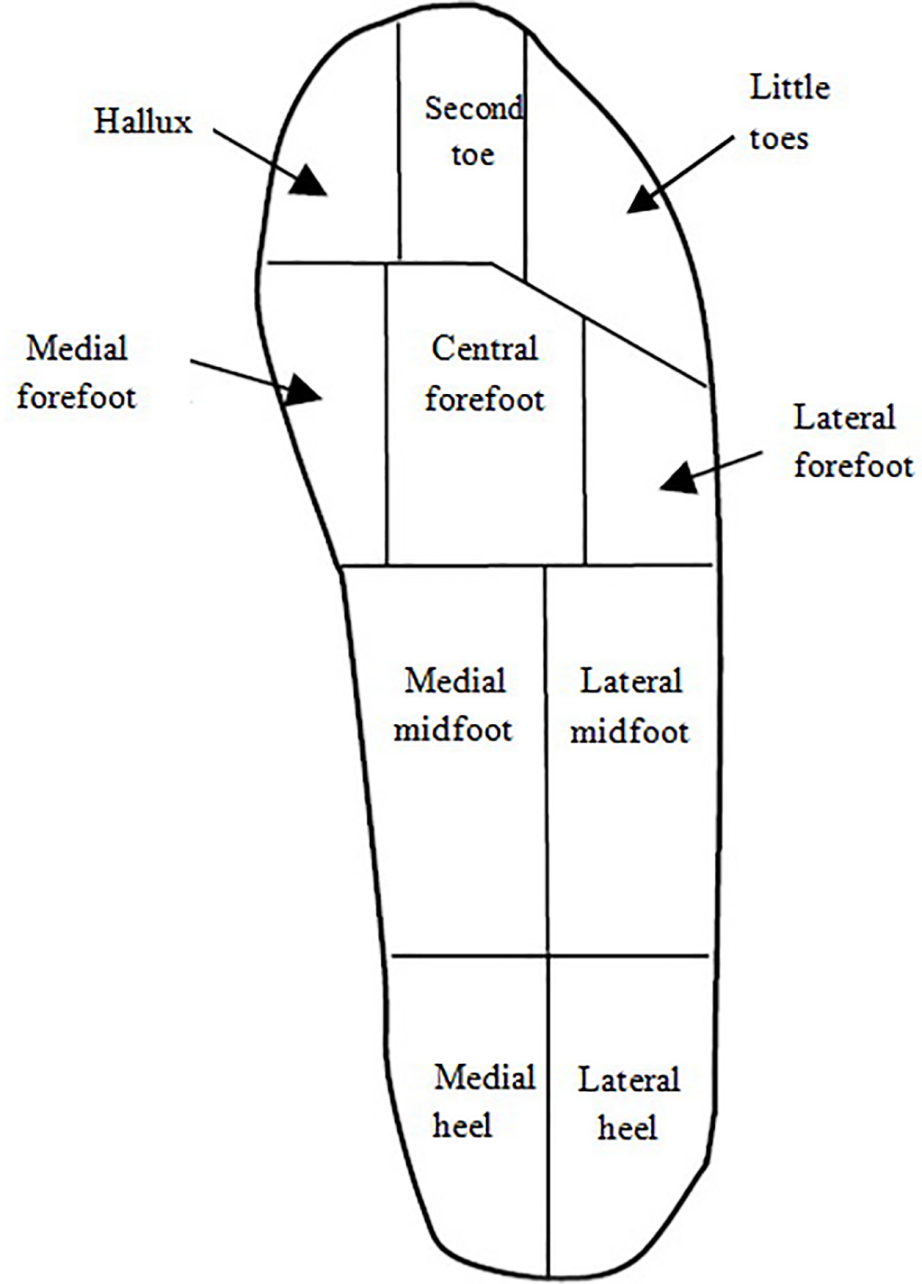
Ten anatomically defined regions of the foot used for plantar pressure measurement.

A regional analysis was performed utilizing ten separate areas of the foot. This analysis was previously done in a similar study [11]. The areas and the density of sensors in each area consisted of the following: medial heel (170), lateral heel (119), medial mid-foot (120), lateral mid-foot (84), medial forefoot (88), central forefoot (22), lateral forefoot (77), hallux (96), second toe (60), and little toes (48) (Fig 1). Peak and mean pressures of the whole foot and each separate area were extracted. Peak and mean pressures are related to the maximum and average loading of anatomical structures at different areas of the foot throughout the movement, respectively. Finally, the mean and peak pressures of the whole foot were calculated. To calculate the mean and peak pressures for each movement, all pressure data were used, regardless of the shoe model (81 measurements) and again for each model, all data were used, not considering the movement (81 measurements).

For statistical analysis, continuous variables were presented as mean (standard deviation); and categorical variables were shown as proportions (percentages). The mean differences in outcome variables, i.e. mean pressure and peak pressure (with 95% confidence intervals) between different shoes and movements were estimated using random-effects generalized least square regression model. If necessary, the outcome variables were log-transformed to normalize the error distribution and stabilize the error variance; the mean differences were anti-logged to derive the geometric means ratio. All analyses were performed using STATA version10 (Stata Corporation, College Station, TX).

## Results

Table 1 shows the mean and peak pressures on the whole foot according to the shoe types and selected movements. Comparative analysis of pressures on the whole foot in three shoe types shows that the mean pressure in Adidas shoes was 11.8% (CI95%: 9.9–14.6%) higher than Lotto and 4.9% (CI95%:2.5–7.2%) higher than Tiger shoes (P<0.001). Furthermore, the mean pressure on the whole foot in penalty kick was 19.6% (CI95%: 17.2–21.9%) lower than shuffle and 18.3% (CI95%: 16–20.9%) lower than sprint tests (P<0.001). Recorded peak pressures on the whole foot in Lotto was 8.8% (CI95%: 4.1–13.6%) and in Tiger, it was 11.8% (CI95%:7–16.7%) higher than Adidas shoes (P<0.001). Moreover, recorded peak pressures on the whole foot in shuffle and sprint tests were 61.1% (CI95%: 56.3–65.9%) and 57.6% (CI95%: 52.8–62.3%) higher than penalty kicks, respectively (P<0.001).

**Table 1:** Mean and peak pressure on the whole foot according to shoe type and movements.

Shoe type	Selected movement	Mean pressure (kPa) [mean(SD)]	Peak presuure (kPa) [mean(SD)]
Adidas	Penalty kick	9.3 (3.8)*	305.9 (73)
	Shuffle	12.4 (8.7)	590.3 (88.9)
	Sprint	10.2 (4)	531.5 (82.4)
Lotto	Penalty kick	7.2 (2.4)	304.1 (38.5)
	Shuffle	10.1 (4.9)	592.1 (66.8)
	Sprint	10.4 (5.1)	528.2 (110.9)
Tigger	Penalty kick	9.2 (4.5)	394.5 (106.4)
	Shuffle	10.7 (3.7)	615.7 (62.9)
	Sprint	10.1 (3.5)	554 (84.6)

* Data shown as Mean (SD)

Also, the mean and peak pressures on each foot area were measured during three movements with 3 shoes (Table 2). Table 2 demonstrates the mean and peak pressure on each foot area during 3 movements. As it is shown in this table, the greatest mean pressures were recorded in forefoot and hallux areas, regardless of the movement or shoe type. Regarding the peak pressure, the highest pressures were measured in medial forefoot in all shoes. Also, relatively high peak pressures were recorded in medial heel, lateral forefoot and hallux. The lowest quantities were measured in mid-foot and little toe areas.

**Table 2:** Mean and peak pressure on different areas of foot according to shoe type and movements.

Area	Shoe			Movement		
	Adidas	Lotto	Tiger	Kick	Shuffle	Sprint
**Mean pressure (kPa)**						
Medial heel	10.4 (7.8)*	7.6 (5.7)	9.7 (9.1)	16.3 (8.7)	3.8 (2.3)	7.4 (3.3)
Lateral heel	5.7 (4.9)	3.9 (3.3)	3.8 (3.5)	7.3 (5)	2.1 (1.8)	4 (2.7)
Medial mid-foot	2.8 (1.8)	2 (1.1)	1.7 (1.5)	3.3 (3)	1.5 (1.2)	1.7 (1.9)
Lateral mid-foot	3.1 (2.1)	3.7 (3.1)	2.6 (1.7)	2.9 (2.8)	3.1 (2.9)	3.4 (3.3)
Medial forefoot	17.8 (10.9)	19.6 (11.8)	22.1 (13.3)	12.3 (9.2)	25.9 (12.3)	21.4 (10.4)
Central forefoot	19.0 (12.5)	19.6 (14.1)	24.2 (15.1)	11.4 (9.7)	27.1 (13.3)	24.4 (13.6)
Lateral forefoot	26.5 (13.6)	25.1 (13.9)	26.9 (14.2)	17.5 (10)	31.9 (13)	29.4 (13.8)
Hallux	12.2 (11.0)	11.2 (9.2)	11.4 (8.2)	6.3 (6.9)	15.7 (8.5)	13 (7.9)
Second toe	8.0 (7.75)	7.7 (6.5)	7.8 (6.9)	1.7 (2.5)	11.1 (8.7)	10.7 (7.7)
Little toes	6.1 (5.2)	5.6 (5.4)	4.3 (3.1)	1.6 (1.5)	7.9 (5.1)	6.6 (4.2)
**Peak pressure (kPa)**						
Medial heel	352.1 (132.6)	320.8 (129.5)	389.4 (145.2)	297.6 (86.7)	363.5 (144.5)	400.2 (154.5)
Lateral heel	247.5 (87.2)	242.8 (121.8)	264.8 (100.1)	202.1 (61.4)	252.1 (105.8)	301.1 (113.6)
Medial mid-foot	153 (69.1)	134.9 (59.9)	133.4 (67.2)	126.7 (62.2)	159.2 (72)	136.2 (59.6)
Lateral mid-foot	150.9 (69.7)	175.4 (68.1)	174.8 (106.7)	131.5 (89.4)	208.2 (71.7)	161.8 (70.9)
Medial forefoot	405 (168.2)	418.6 (181.4)	442.4 (167.6)	237.7 (88.3)	566.2 (97.6)	466.8 (121.9)
Central forefoot	294.8 (144.3)	281.4 (142.1)	333 (163.4)	147.7 (75.7)	415.2 (99.5)	349.4 (117.2)
Lateral forefoot	350.5 (143.4)	346.6 (150.6)	376.5 (137)	211.3 (54.5)	469.6 (108.1)	396.3 (109.4)
Hallux	339.2 (136.3)	336.1 (155.2)	363.1 (139.2)	207.4 (107.1)	453.4 (85.7)	380.9 (105.2)
Second toe	245.3 (127.9)	214.1 (145.8)	252.8 (135)	100.3 (68.2)	310.4 (102.7)	303.6 (111.2)
Little toes	232.5 (91.4)	217.2 (108.8)	208.1 (80.2)	118.4 (52.6)	295.8 (60.4)	246.7 (59.2)

* Data shown as Mean (SD)

Table 3 shows players’ foot areas with highest peak pressure wearing different shoes during movements. This table shows that areas with recorded peak pressure during all three movements are not different between all shoes. This area is medial heel in the case of penalty kick and medial forefoot in cases of shuffle and sprint movements.

**Table 3:** Foot areas with recorded peak pressure during different movements wearing different shoes.

Shoe type	Movement	Foot are with highest peak pressure
Adidas	Penalty Kick	Medial heel
Adidas	Shuffle test	Medial forefoot
Adidas	Sprint test	Medial forefoot
Lotto	Penalty Kick	Medial heel
Lotto	Shuffle test	Medial forefoot
Lotto	Sprint test	Medial forefoot
Tiger	Penalty Kick	Medial heel
Tiger	Shuffle test	Medial forefoot
Tiger	Sprint test	Medial forefoot

As it is demonstrated that the highest pressure in penalty kick test was recorded in the medial heel area. In shuffle and sprint tests, medial forefoot was the area with the highest pressure. These areas were the same using all different shoes.

## Discussion

In this study, we investigated the patterns of pressure distribution inside the shoe during futsal-specific movements using three different shoes. Regarding the novelty, this is the first study investigating the distribution pattern of plantar pressure during the common movements of futsal, as a popular and demanding sport.

The results showed characteristic pressure distribution patterns with specific high pressure areas of foot that corresponded well to the evaluated movements. As stated earlier, the greatest peak pressures in this study were recorded in medial forefoot and medial heel, regardless of shoe or movement type. However, greatest peak pressures were measured in medial heel during penalty kick movement and medial forefoot during sprint and shuffle movements. This pattern was not different among all types of shoes.

Comparison of the recorded mean pressures showed that lower mean pressure on the whole foot was measured in Lotto and then compared to Tiger and Adidas shoes. But the lowest peak pressures were recorded in Adidas, Lotto, and Tiger models, successively. Thus, it seems that mean and peak pressures may not demonstrate significant correlations in each model; as an instance, Adidas showed the lowest peak despite higher mean pressures.

Regarding recorded pressures during three different movements, the highest peak pressure was measured in the shuffle test. It seems that sideward or high speed movements may increase the peak pressures exerted on the foot, as these occur in shuffle and sprint movements.

According to our search, there was no published data for in-shoe pressures during sport-specific movements in futsal. Therefore, the pressure distribution analysis of the evaluated movements should be compared to the pressure data published regarding relatively similar sports such as soccer and running. Comparing pressure distribution measurements with the results presented in the literature warrants particular caution, especially when they were not obtained by the same system. The measuring system has an important role in the pressure distribution values due to the measurement principles as well as the characteristics of the transducers [11,16,24]. It was shown that walking or running speed, shoe design, body mass, sport surface, fatigue, and type of movement influenced pressure distribution [25–31].

In our study, the greatest peak pressures were measured in medial forefoot [467 kPa (122)] and medial heel [400 (155)] during sprint movement; medial heel [298 (87)] and medial forefoot [238 (88)] during kicking movement; and medial forefoot [566 (98)] and lateral forefoot [470 (108)] during shuffle movement. Eils et al. reported the greatest pressures on medial forefoot [595 kPa (171)] and hallux [486 (130) during sprint movement; lateral heel [728 (150)] and medial heel [680 (120)] in ball kicking; and medial heel [655 (145)] and medial forefoot [653 (131)] during cutting movement in soccer shoes [11]. As it is evident, the recorded peak pressures in all foot areas are higher in this soccer study, although this higher pressure may also be due to a stiffer soccer shoe sole that is needed to mount the cleats. Accordingly, Santos showed in a study that peak pressures were higher in soccer shoes in comparison to running shoes, of course, if the test surfaces were similar. So, soccer cleats seem to be responsible for increasing in-shoe plantar foot pressure [32]. Furthermore, relatively higher pressures were recorded in lateral heel in soccer shoes. It is in contrast with our study, in which lateral heel was not among the areas with the highest pressures. This may relate to structural and design differences between two shoe types and the point that probably studs alignment in soccer cleats may shift the pressure to the lateral side. Another study by Carl et al. showed that in running, soccer boots generate excessive foot loadings predominantly under the lateral mid-foot, as compared with running shoes [33]. In a different study on soccer shoes by Wong et al., the highest peak pressures were recorded in medial forefoot, hallux, and medial heel during four soccer-related movements including straight running, sideward cutting, 45° cutting, and jump landing. The authors concluded that compared with straight running at 12 km/h, the peak speed sideward cutting and 45° cutting induced higher peak pressure under the second toe, medial forefoot, medial arch, and medial heel [34]. These results are consistent with our study in which higher peak pressures were recorded on the medial side of foot. Furthermore, the range of recorded pressures is relatively close to that of our study.

The measured peak pressures are comparable to the results reported on running with running or jogging shoes using the same measurement system [25,35]. Chen et al. reported peak pressures under the heel and the medial forefoot of 290 and 390 kPa [25].Weist and Rosenbaum measured values of 250 kPa and 430 kPa under the heel and first metatarsal head during running [35]. Also, Tessuti et al. reported values of 305 and 354 kPa under the medial heel and medial forefoot during running on grass [29].

The comparison of pressure distribution between three different movements (sprint, shuffle, and kicking) shows characteristic patterns that match the evaluated movements. In sprinting, the load is shifted to the forefoot and toes. In shuffle, the load is shifted from the lateral parts of the foot to the medial parts. In kicking, the load is shifted to the heel of the supporting foot. This load transfer leads to increased peak pressures in the mentioned areas.

Of course, this study has some major limitations. First, due to technical problems, we were not able to consider the important parameter of time and synchronize each recorded pressure with different components of gait cycle or specific parts of each movement and just measured the average and peak pressures throughout the movement. However, it seems that the recorded peak pressures are attributed to the most challenging part of each movement. Furthermore, it is sensible that this sample size may not provide sufficient generalization of our results. So, these results should be considered as a preliminary study and other projects with greater sample sizes should be designed accordingly. Moreover, although the foot area in all participants was relatively the same (shoe size 42), we have not assessed the effect of body weight and height in our recorded pressures. However, the relatively narrow range of BMI in participants may attenuate this effect and make it negligible. Finally, it is obvious that skill, speed, and motivation level of each participant may affect the results. Selecting the participants from a relatively similar technical level and motivating them in all movements were made to decrease this effect.

## Conclusions

The results demonstrated characteristic patterns of the pressure distribution in foot during futsal-specific movements. According to these results, the highest pressures were recorded in the medial side of the foot and the type of movement or shoe had a little impact on the results. Furthermore, comparison of our data with comparable studies on soccer and running shoes showed that futsal shoes may have more similarities to running shoes in relation to soccer shoes in terms of pressure distribution patterns. Therefore, as the highest pressures are exerted on the medial side of the futsal shoes, it seems sensible to modify the in-sole design and pay more attention to proper cushioning of the medial side of the shoe. Moreover, foot overuse injuries in futsal may differ from those in soccer and more injuries of overuse are expected in medial parts of the foot.

## Supporting information

S1 Fig Ten anatomically defined regions of the foot used for plantar pressure measurement. (TIF)

S1 Dataset Footpressure. (SAV)

S2 Dataset Analyse. (TXT)
