# Supplementary figures and images for: Comparison of plantar pressure distribution between three different shoes and three common movements in futsal
Source: PLoS One. 2017 Oct 31;12(10):e0187359. doi: 10.1371/journal.pone.0187359 (PMC5663491; doi:10.1371/journal.pone.0187359)

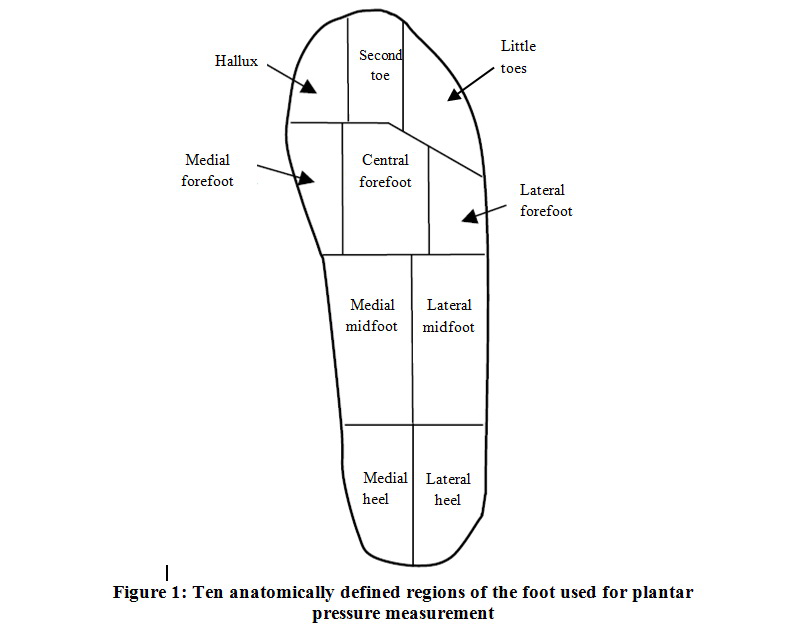

Supplement: S1 Fig — (TIF) [file pone.0187359.s001.tif]
